# Design of Nanocrystalline Suspension of Dutasteride for Intramuscular Prolonged Delivery

**DOI:** 10.3390/nano14221781

**Published:** 2024-11-05

**Authors:** Min Young Jeong, Doe Myung Shin, Min Kyeong Kwon, Ye Bin Shin, Jun Soo Park, In Gyu Yang, Jin Hyuk Myung, Dong Geon Lee, Gi Yeong Lee, Chae Won Park, Ji Won Yeo, Myoung Jin Ho, Yong Seok Choi, Myung Joo Kang

**Affiliations:** College of Pharmacy, Dankook University, 119 Dandae-ro, Dongnam-gu, Cheonan 31116, Chungnam, Republic of Korea; jmy951207@dankook.ac.kr (M.Y.J.); sja9370@dankook.ac.kr (D.M.S.); 72220309@dankook.ac.kr (M.K.K.); shinyb0921@dankook.ac.kr (Y.B.S.); dkjsp0434@dankook.ac.kr (J.S.P.); inq72220320@dankook.ac.kr (I.G.Y.); blueboy0216@dankook.ac.kr (J.H.M.); ldg4337@dankook.ac.kr (D.G.L.); eden1116@dankook.ac.kr (G.Y.L.); 1506chaewon@dankook.ac.kr (C.W.P.); yeo.jiwon13@dankook.ac.kr (J.W.Y.); butable@dankook.ac.kr (M.J.H.)

**Keywords:** dutasteride, nanocrystalline suspension, long-acting injectable, pharmacokinetics, histopathology

## Abstract

The aim of the study is to formulate an injectable nanocrystalline suspension (NS) of dutasteride (DTS), a hydrophobic 5α-reductase inhibitor used to treat benign prostatic hyperplasia and scalp hair loss, for parenteral long-acting delivery. A DTS-loaded NS (DTS-NS, 40 mg/mL DTS) was prepared using a lab-scale bead-milling technique. The optimized DTS-NS prepared using Tween 80 (0.5% *w*/*v*) as a nano-suspending agent, was characterized as follows: rod/rectangular shape; particle size of 324 nm; zeta potential of −11 mV; and decreased drug crystallinity compared with intact drug powder. The DTS-NS exhibited a markedly protracted drug concentration-time profile following intramuscular injection, reaching a maximum concentration after 8.40 days, with an elimination half-life of 9.94 days in rats. Histopathological observations revealed a granulomatous inflammatory response at the injection site 7 days after intramuscular administration, which significantly subsided by day 14 and showed minimal inflammation by day 28. These findings suggest that the nanosuspension system is a promising approach for the sustained release parenteral DTS delivery, with a protracted pharmacokinetic profile and tolerable local inflammation.

## 1. Introduction

Dutasteride (DTS), an inhibitor of 5-alpha reductase types 1 and 2, is prescribed for the management of symptomatic benign prostatic hyperplasia to alleviate symptoms and decrease the risk of acute urinary retention [1]. This potent, competitive, and irreversible aza-steroid compound inhibits the intracellular conversion of testosterone to dihydrotestosterone (DHT), a more potent androgen [2]. A more than 90% reduction in serum DHT levels is observed with oral DTS, whereas only a 70% reduction in serum DHT levels is induced by finasteride [3]. The reduction in serum DHT levels increases epithelial apoptosis, thus decreasing prostate volume and providing relief from the urinary tract symptoms associated with an enlarged prostate [4]. DTS belongs to Biopharmaceutical Classification (BCS) class II compounds, with extremely low water solubility (0.038 ng/mL in water at 25 °C) and high lipophilicity (log*P* value of 6.8) [5]. The oral bioavailability (BA) of the marketed product (Avodart^®^, GlaxoSmithKline, London, UK), which comprises solubilized DTS in a mixture of monoglycerides and diglycerides of caprylic/capric acid, is still unsatisfactory at about 40–60% in humans [6]. Considering that DTS requires long-term use to achieve therapeutic effects [7], a prolonged parenteral delivery system offering an effective therapeutic level for several weeks following a single administration, thereby requiring extended dosing intervals, is desirable. In a previous study, a polylactic-co-glycolic acid (PLGA) biodegradable polymeric microparticle system was designed for the prolonged delivery of DTS, providing a protracted pharmacokinetic (PK) profile over 4 weeks. However, it was quite challenging to administer a sufficient DTS dose because of the low drug loading in the particles with a complicated fabrication process [8].

Long-acting parenteral drug delivery systems are increasingly exploited to enhance the management of chronic conditions by reducing the need for daily medication regimens in diseases such as HIV/AIDS, psychiatric disorders, cancer, and diabetes. These systems, such as aqueous drug suspensions, polymeric microsphere or core-shell, oil-based injectables, liposomes, and in situ-forming implant-based depots, afford controlled drug release at injection sites principally by providing a physical barrier or through the intrinsic low aqueous solubility of the drug [9,10,11,12,13,14,15]. Drug nanocrystalline suspensions (NSs) have emerged as a promising tool for designing prolonged parenteral delivery systems for insoluble pharmaceuticals [10,16,17,18]. An NS is a colloidal dispersion of nanosized drug particles stabilized by a minimal quantity of polymeric and surfactant stabilizers in the continuous phase [19]. The parenteral delivery system provides high drug loading, excellent pharmacokinetic persistence, and ease of scale-up. Following intramuscular (IM) injection, drug particles gradually dissolve at the injection site and partition into the bloodstream, providing a continuous drug concentration profile over several weeks [16,20]. Moreover, drug particle aggregation and subsequent foreign body reactions, including granulomatous inflammatory reactions at the injection site, macrophage infiltration, phagocytosis of the injected dose, fibrosis, and angiogenesis, affect drug dissolution patterns at the injection site, modulating the pharmacokinetic profile following IM injection [21]. With several advantages of NS-based long-acting injectable (LAI) systems, NS products like Invega Sustenna^®^ (paliperidone palmitate), Invega Trinza^®^ (paliperidone palmitate), Abilify Maintena^®^ (aripiprazole), and Cabenuva^®^ (cabotegravir + rilpivirine) have been commercialized [10,22]. However, to the best of our knowledge, no attempts have been made to formulate an NS-based LA system for DTS.

Therefore, our objective was to design an NS system for DTS and evaluate its feasibility as an LA delivery system for in vivo pharmacokinetic evaluations. In this study, lab-scale bead milling was employed to fabricate DTS-loaded nanocrystalline suspensions (DTS-NSs). The prepared DTS-NSs were characterized based on their particle size, surface charge, morphology, and crystallinity. Additionally, the pharmacokinetic profile of DTS was assessed in rats using a validated liquid chromatography-tandem mass spectrometry (LC-MS/MS). Moreover, the degree of local inflammatory response at the injection site was histologically observed following a single IM administration.

## 2. Materials and Methods

### 2.1. Materials

The DTS drug powder (purity > 98% *w*/*w*) was obtained from MSN Laboratories Private Ltd. (Hyderabad, India). Sodium carboxymethyl cellulose (Na. CMC) with an average molecular weight of 90,000 g/mol (degree of substitution of 0.7, product number 419273), tyloxapol, polyethylene glycol 4000 (PEG 4000), formic acid, and 10% (*v*/*v*) neutral-buffered formalin were purchased from Sigma Chemical (St. Louis, MO, USA). Macrogol (15)-hydroxystearate (Solutol HS15), polyoxyl-35 castor oil (Cremophor ELP), polyvinylpyrrolidone K17 (PVP K17), and polyoxyethylene (160)-polyoxypropylene (30) glycol (Poloxamer 188) were provided by CTC Bio (Hwaseong-si, Gyeonggi-do, Republic of Korea). Polysorbate 20 (Tween 20) and polysorbate 80 (Tween 80) were obtained from Croda Korea (Seongnam-si, Gyeonggi-do, Republic of Korea). Mannitol was purchased from Roquette Frères (Lestrem, France). Finasteride (≥99.0%), which served as the internal standard (IS), formic acid (LC-MS grade), and sodium hydroxide (reagent grade) were purchased from Sigma Aldrich (St. Louis, MO, USA). Water, acetonitrile, methanol, methyl *tert*-butyl ether (MTBE), and dichloromethane were obtained as HPLC grade from J. T. Baker (Phillipsburg, NJ, USA).

### 2.2. Preparation of DTS-Loaded NSs Using Bead-Milling Technology

**Figure 1:**
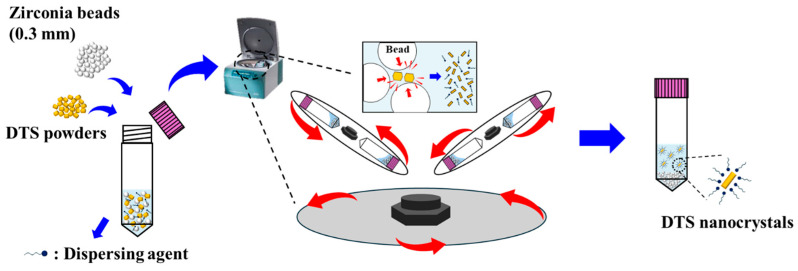
Illustration of DTS-NS manufacturing process using dual centrifugation-based bead-milling technique.

DTS-NS was prepared using a dual-centrifugation-based bead-milling technique (Figure 1). Dual centrifugation-based bead milling was conducted using a Zentrimix 380R (Andreas Hettich GmbH und Co KG, Tuttlingen, Germany). An isotonic aqueous vehicle (286 mOsm/kg) was prepared by adding 50 mg/mL of mannitol to distilled water. Suspending agents (Cremophor ELP, Cremophor HS15, Tyloxapol, Tween 20, Tween 80, Na. CMC 90 K, PEG 4000, and PVP K17) were dissolved in an isotonic solution at a concentration of 2.5–10 mg/mL using a multivortex (Multi Reax, Heidolph, Schwabach, Germany). DTS powder (40 mg/mL) and 1 g of zirconium dioxide beads (average diameter 0.3 mm) were added to the milling tube, followed by the addition of 1 mL of aqueous vehicle. The mixtures were pre-wetted for 5 min using a vortex mixer (Vortex-Genie 2; Scientific Industries, Chicago, IL, USA) at room temperature. Coarse dispersions in the milling tube were bead milled at different centrifugation speeds (500, 1000, and 1500 rpm) for 1 h. During bead-milling process, the cooling device in instrument was set to −10 °C to prevent the potent thermal degradation of DTS during the milling process. NS were collected using a 26 G syringe and placed in a scintillation vial at 25 °C for further experiments.

### 2.3. Morphological and Physical Characterizations of DTS-NSs

#### 2.3.1. Morphological Observation

The morphological features of the DTS raw material and DTS-NS were observed using scanning electron microscopy (SEM; Merlin Gemini II, Carl Zeiss Inc., Jena, Germany). For SEM observation, DTS-NS was centrifuged at 4000 rpm, 20 °C for 10 min to remove the supernatant and obtain the nanoparticles. The DTS-NS particles were dried at room temperature in a desiccator for 24 h. The dried samples were mounted on aluminum stubs with carbon tape and sputtered at 15 mA using a sputter coater (Model 108AUTO, Cressington, Liverpool, UK). The SEM observations were conducted at an accelerating voltage of 5 kV.

#### 2.3.2. Particle Size and Zeta Potential

The mean particle size and polydispersity index (PDI), which indicate the uniformity of the size distribution, were determined using dynamic light scattering (DLS) technique [23,24]. Approximately 100 μL of uniformly dispersed NS was diluted 100-fold with distilled water (DW) at room temperature for 5 min and transferred to a disposable cuvette. Analysis was conducted using a Zetasizer Nano^®^ ZS instrument (Malvern Instruments, Malvern, UK) with a 4 mW He-Ne laser (633 nm) at a 173° back-scattering angle. All measurements were performed in triplicate at 25 °C.

#### 2.3.3. Solid-State X-Ray Diffraction (XRD) Pattern

The solid-state crystallinity of the drug nanocrystals in the NS samples was analyzed using an X-ray diffractometer (Ultima IV; Rigaku Corporation, Tokyo, Japan) [25]. DTS-NS was diluted 10-fold with DW and then centrifuged at 4000 rpm for 10 min at 20 °C. The supernatant was removed, and the remaining drug nanocrystals were collected and dried in a desiccator for 24 h. Approximately 30 mg of the dried sample was placed in the holder and analyzed under Cu-K radiation at 40 kV and 40 mA, with a scanning range of 5°–45°, scan steps of 0.02°, and a scan speed of 2 °/min. The crystallinity of DTS-NS was calculated from XRD data. The calculations were performed using OriginPro 2023 software (OriginLab, Northampton, MA, USA). The crystallinity index was determined as the ratio of the area under all crystalline peaks to the total area of the diffraction spectrum. The areas of the identified crystalline peaks and the total diffraction spectrum were calculated based on the processing baseline and peak lines defined in the diffraction profile [26].

#### 2.3.4. Differential Scanning Calorimetry (DSC) Analysis

Samples (approximately 2 mg) collected using the above methods were loaded into a standard aluminum pan and hermetically sealed. The samples were heated at a rate of 10 °C/min from 40 to 290 °C. An inert atmosphere was ensured by continuous purging with nitrogen gas at a flow rate of 20 mL/min, with an empty aluminum pan serving as the reference standard.

### 2.4. In Vitro Dissolution Test

The in vitro dissolution profile of DTS from the NS formulation was assessed under sink conditions by adding Cremophor ELP (2% *w*/*v*) to 10 mM sodium phosphate buffer (pH 7.4). DTS drug powder and DTS-NS, each containing 2 mg of DTS, were immersed in 220 mL of dissolution medium, maintained at 37 ± 0.5 °C, and stirred at 100 rpm using a shaking dissolution tester (Model BF-60SIR; Biofree, Seoul, Republic of Korea). At predetermined intervals, 1 mL aliquots were withdrawn and centrifuged at 13,000 rpm for 10 min to remove any insoluble particulates. The volume of the dissolution medium was kept constant by replenishing it with an equivalent volume of prewarmed fresh medium (37 °C). The DTS concentrations in the samples were quantified using an Agilent high-performance liquid chromatography (HPLC) system equipped with a pump (G1212B), ultraviolet-visible (UV-VIS) detector (G1314B), degasser (G1379B), column oven (G1316B), and autosampler (G1367B). The mobile phase consisted of a 45:55 (*v*/*v*) mixture of distilled water and acetonitrile, which was passed through a C18 column (4.6 mm × 150 mm, 5 μm; Phenomenex, USA) at a flow rate of 1 mL/min. The injection volume and column temperature were set at 20 μL and 30 °C, respectively. The UV-VIS detector wavelength was set to 210 nm and the retention time of DTS was recorded as 5.8 min. The calibration curve for DTS, within the concentration range of 0.1 to 40 μg/mL, was linear (R^2^ = 1.0000, Y = 27.173X + 2.2967). The limit of detection (LOD) and limit of quantitation (LOQ) for the analytical method were determined to be 0.01 and 0.05 μg/mL, respectively.

### 2.5. In Vivo Pharmacokinetic Evaluation in Rats

The plasma concentration–time profiles of DTS following IM DTS-NS injection or intravenous injection of the drug solution were comparatively evaluated in rats with the approval of the Institutional Animal Care and Use Committee (IACUC) of Dankook University (approval number: DKU-23-033). Thirteen-week-old male Sprague–Dawley rats (400 ± 40 g) were obtained from Samtako (Osan-si, Gyeonggi-do, Republic of Korea) and housed in a controlled animal room (23 ± 1 °C and 50 ± 5% relative humidity) under a 12 h light–dark cycle.

For the in vivo pharmacokinetic study, rats were randomly divided into two groups (n = 5 per group) after a 3-day acclimatization period. Uniform DTS-NS was administered intramuscularly to the gastrocnemius muscles of the hind leg at a dose of 5 mg/kg (DTS) using a 26 G syringe. For IV injection, 5 mg of DTS was dissolved in 80% *v*/*v* DMSO (in saline) and administered via the rat’s tail vein at a dosage of 0.2 mg/kg (as DTS) over approximately 30 s using a 26 G syringe. Following the administration, the rats were allowed free movement in the cage with access to water and standardized chow. Blood samples (approximately 0.7 mL) were collected from the jugular vein at predetermined intervals (for IM NS: 0.04, 0.08, 0.17, 0.33, 0.50, 1, 2, 4, 7, 14, 21, 28, 42, and 56 day; for IV drug solution: 0.0035, 0.01, 0.021, 0.042, 0.083, 0.17, 0.33, 0.50, 1, 2, and 4 day) using a 26 G heparinized syringe (50 IU heparin). Plasma was separated by centrifugation at 13,000 rpm for 10 min at 4 °C within 1 h of collection and stored at −80 °C.

The levels of DTS in rat plasma were determined using a modified version of a previously developed method involving liquid-liquid extraction (LLE) and liquid chromatography-tandem mass spectrometry (LC-MS/MS) [27]. Briefly, 50 μL of rat plasma was mixed with 100 μL of an aqueous sodium hydroxide solution (1 mol/L) and 600 μL of an LLE extraction reagent (MTBE:dichloromethane, 70:30, *v*/*v*) containing 1 ng/mL of the IS for 10 min. After centrifugation of the mixture for 5 min at 25 °C, the LLE extraction reagent layer was dried under a nitrogen stream. The residue was reconstituted in 200 μL of a 50% aqueous methanol solution, and 5 μL of the resulting solution was analyzed using a Shimadzu Nexera UPLC system (Tokyo, Japan) and a Shimadzu LCMS 8050 triple quadrupole mass spectrometer interfaced through ESI in positive ion mode. For separation, a Phenomenex Gemini NX-C18 column (2.0 × 150 mm, 3 μm, Torreace, CA, USA) was employed along with an isocratic mobile phase (0.1% aqueous formic acid solution:acetonitrile, 30:70, *v*/*v*). The sample was separated over eight minutes at a flow rate of 0.25 mL/min. For multiple reaction monitoring (MRM) of DTS, 529.3 *m*/*z* (precursor ion)/461.1 *m*/*z* (product ion)/−25 V (collision energy), 529.3 *m*/*z*/365.0 *m*/*z*/−32 V, and 529.3 *m*/*z*/236.0 *m*/*z*/−56 V were used as the screening transition, the confirmatory transition 1, and the confirmatory transition 2, respectively. Additionally, for the IS, the screening transition of 373.2 *m*/*z*/317.4 *m*/*z*/−19 V, the confirmatory transition 1 of 373.2 *m*/*z*/305.1 *m*/*z*/−27 V, and the confirmatory transition 2 of 373.2 *m*/*z*/72.0 *m*/*z*/−43 V were applied.

Pharmacokinetic parameters such as area under the plasma concentration versus time curve from 0 to 56 days (AUC_0–last_), from 0 to infinity (AUC_0–inf_), maximum plasma concentration (*C*_max_), time to peak maximum plasma concentration (*T*_max_), distribution half-life (*T*_1/2α_), and elimination half-life (*T*_1/2β_) were calculated using WinNonlin^®^ version 5.2 (Pharsight Co., Mountain View, CA, USA).

### 2.6. In Vivo Local Inflammatory Responses at Injected Sites Following IM Injection

In vivo local inflammatory responses at the injected gastrocnemius muscle following IM injection of DTS-NS or normal saline were assessed in thirteen-week-old male Sprague–Dawley rats weighing 400 ± 40 g. Rats received IM injections (5 mg/kg per site, which is equivalent to PK study) into both gastrocnemius muscles of the hind legs, while those in the control groups were administered equal volumes of normal saline. Gastrocnemius muscle tissues were surgically isolated at predetermined time points (7, 14, and 28-days post-dosing) and subjected to previously reported tissue treatment procedures [28]. Briefly, isolated muscle tissues were fixed in 10% paraformaldehyde for 72 h at 4 °C and underwent routine processing for histological analysis. Paraffin-embedded tissue specimens were sectioned into 10 μm thickness using a microtome (Model Leica RM2165, Wetzlar, Germany), followed by deparaffinization with two steps of xylene and dehydration through descending ethanol grades (100%, 90%, 80%, and 75%). Subsequently, the sections were stained with hematoxylin and eosin (H&E) for histological examination.

### 2.7. Statistical Analysis

The experimental data were analyzed using a one-way analysis of variance (ANOVA), followed by post hoc multiple comparisons using SPSS software (version 23; SPSS Inc., Chicago, IL, USA). Statistical significance was set at *p* < 0.05.

## 3. Results and Discussion

### 3.1. Screening of Suspending Agent to Formulate DTS-NS System

Dual-centrifuge bead milling is an advanced method for producing nanoparticle suspensions that is known for its rapid and effective particle size reduction, often achieving sizes comparable to or smaller than those obtained through traditional wet milling methods [29,30]. The Zentrimix system exemplifies this technique by featuring a unique design that facilitates simultaneous milling and separation processes. This enhances the interaction between the milled beads and the drug particles, leading to improved dispersion stability. In addition, Zentrimix can process multiple small batches simultaneously, significantly reducing the processing time and increasing the throughput [30,31].

The drug concentration in the NS samples was 40 mg/mL. Considering the oral daily dose of DTS (0.5 mg per day), the injection of 0.5 mL of NS formula (40 mg/mL) provides approximately 40 days of drug dosing following a single administration. In suspension formulations, the selection of a suspending agent is a critical process that affects drug crystal size and homogeneity, as well as physical stability under storage conditions [32,33]. Therefore, different polymeric and amphiphilic pharmaceutical excipients were screened to formulate a stable DTS-NS system.

**Figure 2:**
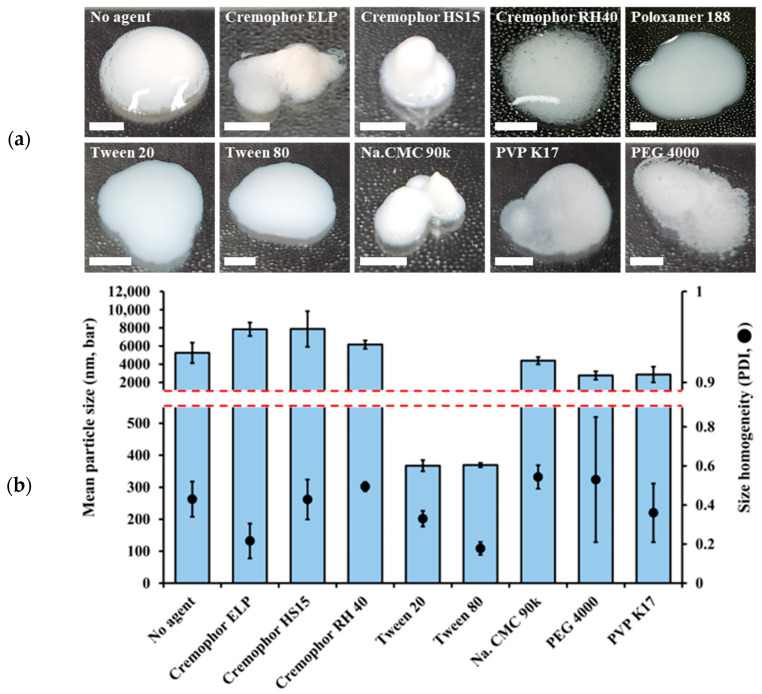
Influence of suspending agents on (**a**) the appearance and (**b**) particle size and homogeneity of DTS-NS formulas prepared using a lab-scale bead-milling technology. Notes: (**a**) All images were captured on glass slides within 10 min after fabrication (scale bar: 5 mm). (**b**) The concentrations of DTS and suspending agent were fixed at 40 mg/mL and 5 mg/mL, respectively. Bead milling was carried out at 1500 rpm for 1 h under conditions of −10 °C. Data represent means ± SD (n = 3).

At first, the effect of the kind of suspending agent on the appearance and particle size of DTS-NS was evaluated at the drug and suspending agent concentration of 40 and 0.5 mg/mL, respectively, with a bead milling speed of 1500 rpm for 1 h. Except for Tween 20 and 80, the employed suspending agents could not evenly distribute the hydrophobic DTS particles in the aqueous vehicle, and no NS systems smaller than 2 µm were formed without uneven distribution or particle agglomeration. Conversely, NS prepared with Tween 20 and Tween 80 exhibited evenly distributed drug particles without agglomeration (Figure 2a). The drug particle size and dispersion homogeneity of NSs prepared using different suspending agents are shown in Figure 2b. Drug particles prepared using Cremophor ELP, Cremophor HS 15, Cremophor RH 40, Na. CMC 90 K, PEG 4000, and PVP K17 were determined to measure over 1 µm. In contrast, the drug particle sizes in Tween 20 and Tween 80 were 367 and 369 nm, respectively. Moreover, the PDI values of NS prepared using Tween 20 and Tween 80 were determined to be 0.33 and 0.18, respectively, indicating a more homogeneous size distribution for Tween 80 than for Tween 20 (Figure 2b). Tween 80 is characterized by its molecular structure, which includes a hydrophilic polyoxyethylene chain and a hydrophobic fatty acid tail derived from oleic acid [34]. This combination resulted in a longer fatty acid chain than Tween 20, thereby providing enhanced stability and reducing the likelihood of phase separation. Additionally, Tween 80 provides steric hindrance, which effectively inhibits particle aggregation by forming a protective layer around the drug particles. Such structural characteristics are essential for maintaining the integrity of the drug over extended periods because they facilitate the formation of a stable emulsion or suspension. Based on these findings, Tween 80 was selected as the suspending agent for preparing DTS-NS.

### 3.2. Effects of Tween 80 Concentration on Drug Particle Size and Homogeneity of DTS-NS

The concentration of the suspending agent significantly affected the surface free energy and thermodynamic behavior of the colloidal system. An appropriate ratio of the drug to the suspending agent can reduce particle size, enhance dispersion stability, decrease particle sedimentation rate, and facilitate redispersion [32,35]. However, excessively high concentrations of the suspending agent may cause drug solubilization, particle agglomeration, and subsequent precipitation [36].

**Figure 3:**
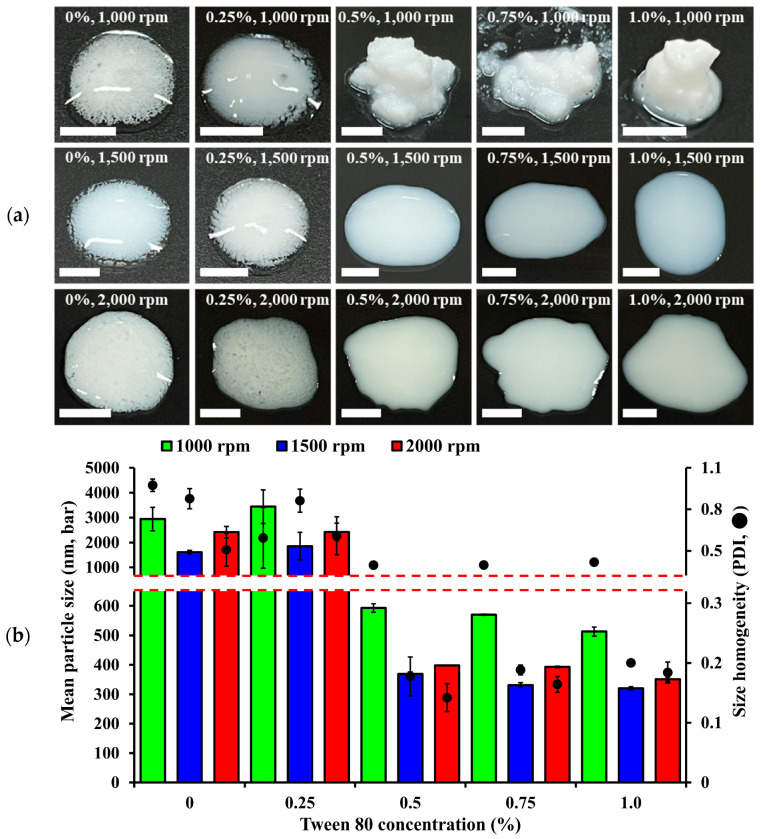
Effect of the concentration of Tween 80 on the crystal size of DTS-NSs prepared using a lab-scale bead-milling technology. (**a**) The appearance of drug suspension prepared with different Tween 80 concentrations. (**b**) Effect of Tween 80 concentration on mean particle size and homogeneity. Notes: (**a**) All images were captured on glass slides 10 min after preparation (scale bar: 5 mm). (**b**) Concentrations of DTS and Tween 80 were set at 40 and 0–10 mg/mL, respectively. Bead milling was performed at 1000–2000 rpm for 1 h under conditions of −10 °C. Data are presented as means ± SD (n = 3).

As shown in Figure 3a, NS prepared with a low quantity of Tween 80 (0 or 2.5 mg/mL) showed particle agglomeration, regardless of milling intensity. At a milling speed of 1000 rpm (milling time of 1 h), NSs with 0.5, 0.75, and 1.0% Tween 80 were uniformly dispersed within the initial 5 min but were aggregated and precipitated within 10 min. Conversely, well-dispersed uniform NSs were prepared at milling speeds of 1500 and 2000 rpm with Tween 80 concentrations of 0.5, 0.75, and 1.0%. This indicates that lowering the surface tension with a suspending agent, along with adequate pulverization energy, is necessary for wet bead milling to achieve a uniform, submicron-sized drug particle dispersion. The drug particle size and homogeneity of the DTS-NS were further assessed (Figure 3b). When the concentration of Tween 80 was set above 0.5%, the drug particle size tended to decrease drastically, and at a milling speed of 1000 rpm, the particle sizes obtained with Tween 80 concentrations of 0, 0.25, 0.5, 0.75, and 1.0% were determined to be 2940, 3440, 592, 570, and 513 nm, respectively. At milling speeds of 1500 rpm and 2000 rpm for 1 h, the particle sizes for Tween 80 concentrations of 0, 0.25, 0.5, 0.75, and 1.0% were 1612, 1853, 369, 332, and 320 nm, and 2418, 2425, 398, 393, and 351 nm, respectively. Irrespective of the milling speed, Tween 80 concentrations of 0.25% or lower resulted in particle sizes in the micrometer range. Conversely, when the Tween 80 concentration was 0.5% or higher, nanoparticles smaller than 1 µm were observed (Figure 3b). These results indicate that a minimum amount of surfactant is required to produce nanosuspensions, regardless of the milling speed. Nanosuspensions with particle sizes below 1000 nm have an exponentially larger surface area than microsuspensions [37]. Therefore, to stabilize the extensive surface area of nanosuspensions, an adequate amount of surfactant is required [38].

### 3.3. Morphological and Physical Characteristics of DTS-NS

**Figure 4:**
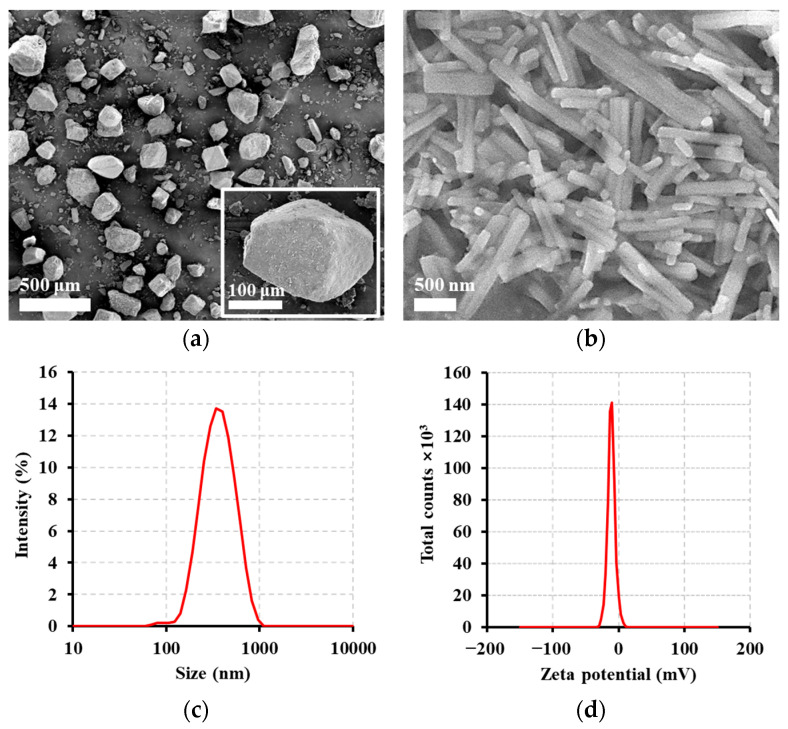

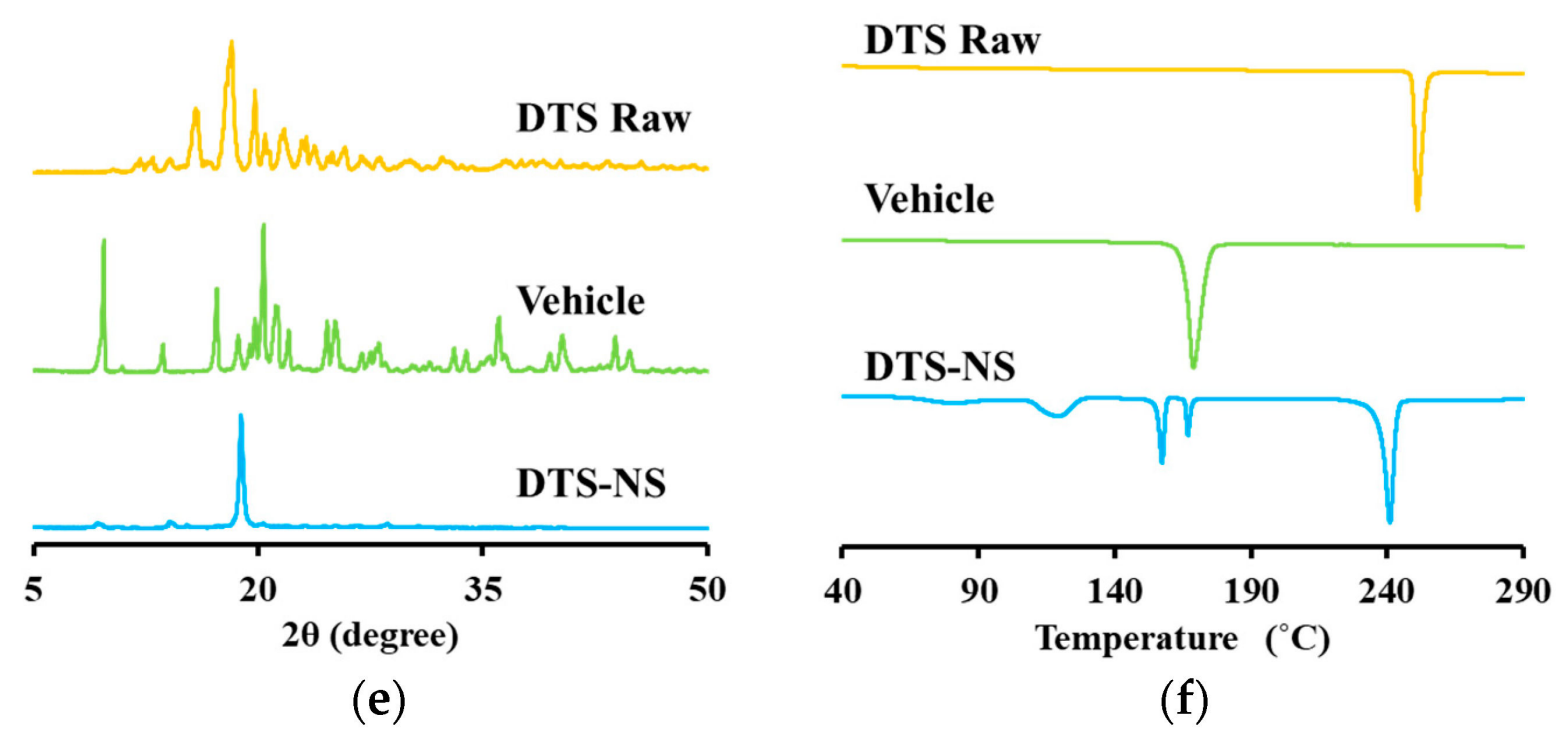
Morphological and physical characteristics of DTS-NS. Scanning electron microscopic (SEM) image of (**a**) raw material and (**b**) DTS-NS. (**c**) Particle size distribution and (**d**) zeta potential of DTS-NS. Representative (**e**) XRD patterns and (**f**) DSC curves of the raw material, aqueous vehicle, and DTS-NS.

SEM observations revealed marked differences in the morphology between the DTS raw material and DTS-NS. The raw material exhibited rhombohedral shapes with diameters of 102–352 µm (Figure 4a). However, the particle size markedly decreased after bead milling, accompanied by a change in the morphology to rod-shaped crystals (Figure 4b). The particle size of DTS-NS was determined to be 324 nm (Figure 4c), with a zeta potential value of −11.1 mV (Figure 4d). The size of DTS-NS was within the size range of commercialized LAI drug suspensions. The mean crystal sizes of paliperidone palmitate (Invega Sustenna^®^, Janssen, NJ, USA), aripiprazole (Abilify Maintena^®^, Otsuka Pharmaceutical Inc., Tokyo, Japan), olanzapine (Zyprexa Relprevv^®^, Eli Lilly, Indianapolis, IN, USA), and rilpivirine suspensions (Cabenuva^®^, ViiV Healthcare, London, UK) have been reported to be <1 µm [39], 1–10 µm [40], 1–13 µm [41], and <0.5 µm [42], respectively. The optimized DTS-NS system was physicochemically stable, maintaining its drug content, particle size, and uniformity over 4 weeks at 25 °C (Table S1).

The solid-state crystallinity of DTS in the NS formula was further evaluated using XRD and DSC. The solid-state crystallinity of DTS in the NS formula was evaluated using XRD and DSC. The XRD pattern of the DTS raw material showed characteristic peaks at 15.8°, 18.2°, 19.8°, 20.5°, and 21.7°, with the crystallinity index of 99.3%. However, in DTS-NS, most peaks were not observed except for a peak at 18.9°, with a crystallinity index of 83.7% (Figure 4e). The disappearance and formation of different peaks in the XRD pattern indicate a change in the crystal form [43,44]. DSC measurement also supported the XRD data with a shift in endothermic peak at 251.2 °C to 241.2 °C (Figure 4f). The decrease in the melting point of DTS-NS compared with that of raw DTS is attributed to the weakened drug crystallinity caused by particle size reduction. The endothermic peaks at 157.4 and 166.9 °C in DTS-NS are presumed to have originated from residual Tween 80 and D-mannitol [45,46].

### 3.4. In Vitro Dissolution Profile of DTS-NS

**Figure 5:**
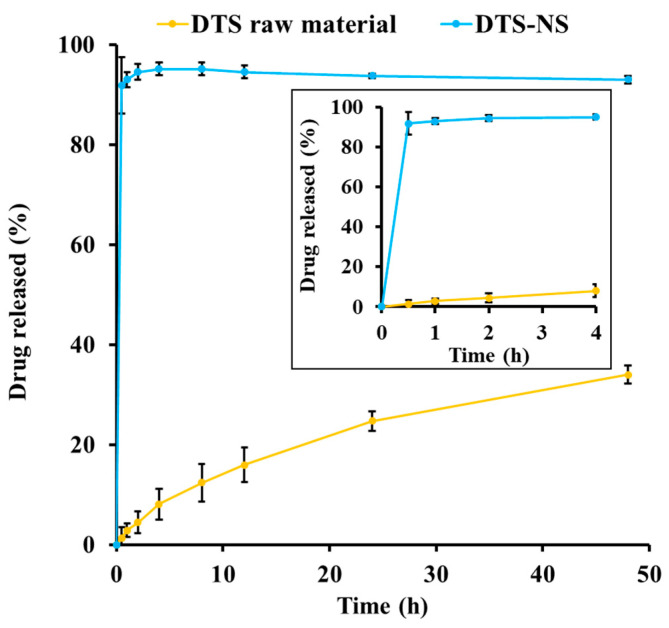
In vitro dissolution profile of DTS raw material and DTS-NS under sink condition. The sink condition was guaranteed by adding 2% *w*/*v* Cremophor EL to 10 mM sodium phosphate buffer (pH 7.4). Note: Data represent mean ± SD (n = 3).

Figure 5 shows the dissolution profile of DTS-NS under sink conditions. Cremophor EL was added to phosphate-buffered saline to provide sufficient water solubility of DTS by incorporating a hydrophobic compound into the micelle structure above the critical micelle concentration [47]. The equilibrated DTS solubility in 2% *w*/*v* Cremophor EL-containing PBS was determined to be 30 μg/mL, which is adequate solubility to provide a sink condition. As expected, the dissolution of DTS from the NS system was significantly faster than that of the raw material. The NS system provided >90% drug release after 0.5 h, whereas that of the raw material was determined to be 1.3%. This dissolution pattern can be explained by the Noyes–Whitney equation: dM/dt = k·S·Cs, where dM/dt is the dissolution rate, k is the rate constant, and S is the surface area of the drug particle, and Cs is the drug solubility. Drug particle size reduction led to a marked increase in the surface area, thereby promoting the dissolution of DTS in aqueous media. Moreover, the reduction in particle size promoted drug dissolution with weakened crystallinity, as observed in the XRD and DSC experiments.

### 3.5. Establishment of LC-MS/MS Analysis Method for Determination of DTS in Plasma

**Figure 6:**
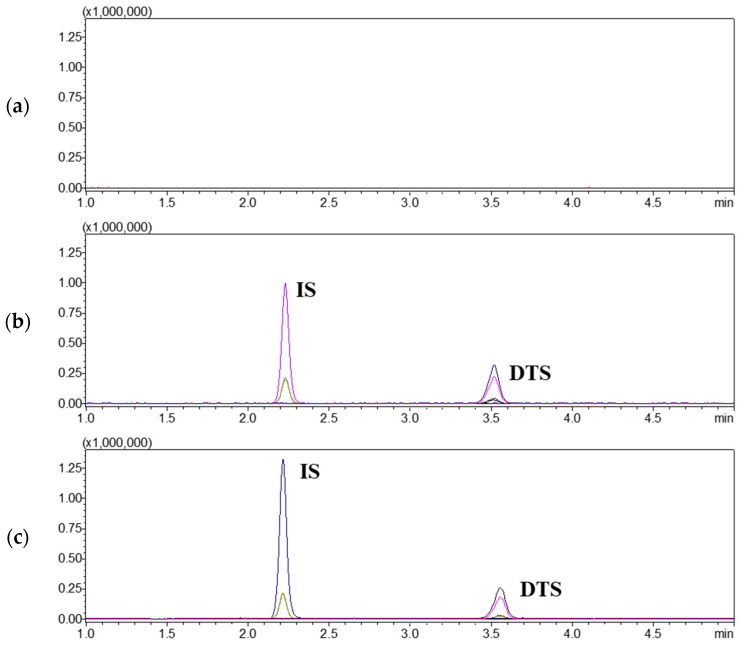

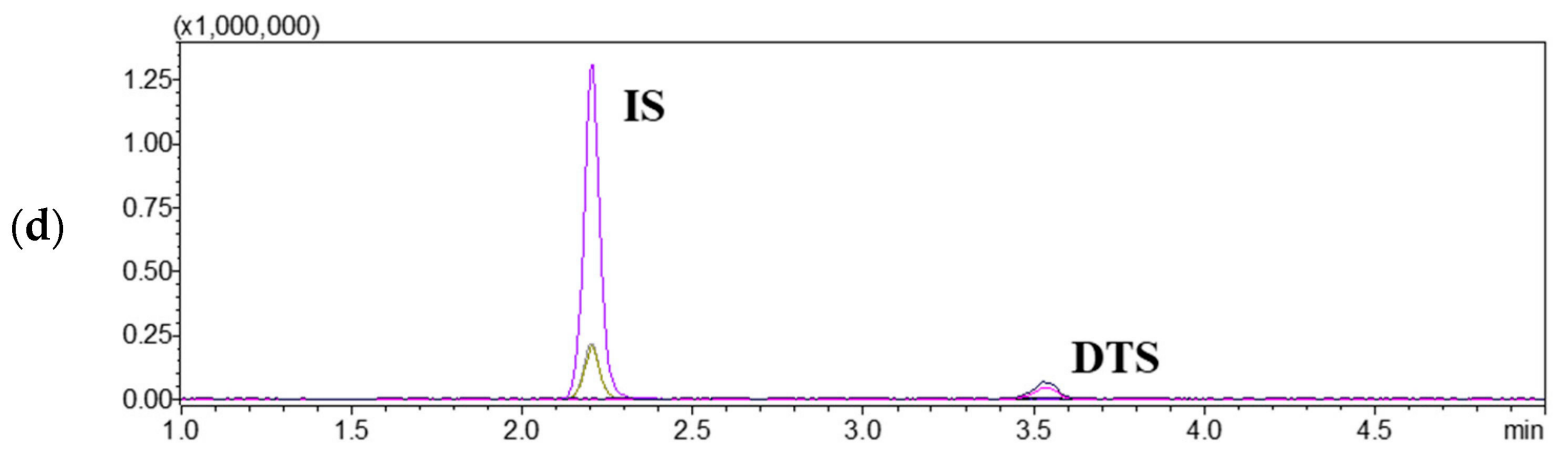
Multple reaction monitoring chromatograms of double blank rat plasma (**a**), 50 ng/mL of dutasteride-spiked rat plasma (**b**), rat plasma taken five minutes after dutasteride intravenous (IV) administration (**c**), and rat plasma taken eight hours after dutasteride IV administration (**d**). DTS and IS stand for dutasteride and finasteride (internal standard), respectively.

While our research team previously developed a method to determine DTS in rat plasma, it had some limitations: the use of a relatively large volume of plasma (100 μL) and limited sensitivity (a lower limit of quantitation (LLOQ) of 12.10 ng/mL) [27]. Since these limitations were due to the use of a low-grade mass spectrometer with low sensitivity, the method was modified to be compatible with a higher-grade mass spectrometer with higher sensitivity. Thus, the volume of plasma for LLE was reduced to 50 μL to prevent the contamination of the higher-grade mass spectrometer. Generally, the reduction in sample volume negatively affects the sensitivity of the method, but the use of the higher-grade mass spectrometer with higher sensitivity in the modified method more than compensated for this: the LLOQ improved to 0.1 ng/mL. While the higher limit of quantitation was reduced from 400 ng/mL to 50 ng/mL, all in vivo evaluation results in the present study were within the dynamic range of the modified method (0.1–50 ng/mL) (Figure 6). The original composition of the LLE extraction reagent (MTBE:dichloromethane, 70:30, *v*/*v*) was retained in the modified method. Considering the use of 100% MTBE as the LLE solvent to extract donepezil (log*P* of 4.3) and rotigotine (log*P* of 4.9) in rat plasma, the use of an LLE solvent with slightly more non-polar properties by adding dichloromethane to 30% (*v*/*v*) rather than 100% MTBE to extract DTS (log*P* of 6.8) in rat plasma seems very reasonable [5,48,49,50,51]. The modified method was successfully validated in terms of selectivity, sensitivity, matrix effect, linearity, accuracy, precision, recovery, and stability following FDA guidelines [52].

### 3.6. In Vivo Pharmacokinetic Evaluation of DTS-NS in Rats

**Figure 7:**
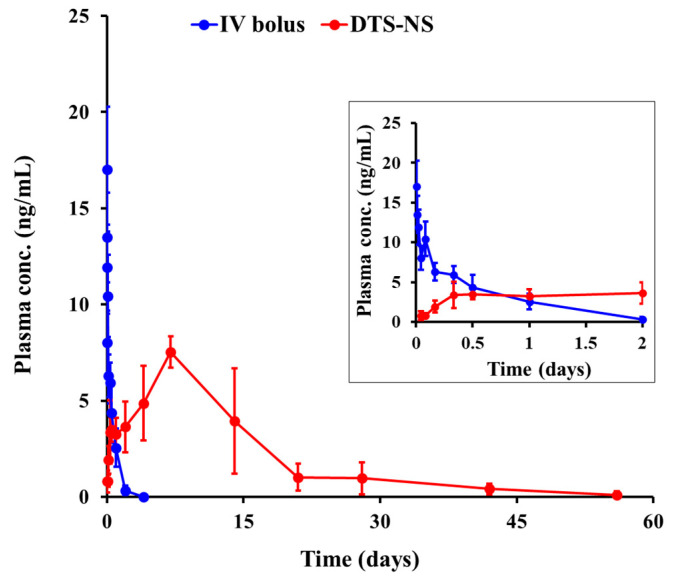
The plasma concentration–time profiles of DTS following IM injection (5 mg/kg as DTS) of DTS-NS and IV injection (0.2 mg/kg as DTS) of DTS solution in rats. Note: Data represent mean ± SD (n = 5).

The pharmacokinetic profile of DTS following IM injection of NS or IV injection of the drug solution was evaluated in rats (Figure 7). Parameters calculated from the pharmacokinetic profiles are listed in Table 1. Following IM injection, the drug concentration in the plasma gradually increased, reaching a peak level 5–10 days after dosing (T_max_ = 8.40 days). The C_max_ value of IM NS was determined to be 8.06 ng/mL. This suggests that despite the rapid dissolution of NS under in vitro sink conditions, the drug nanocrystals slowly dissolved and were absorbed at the injection site in rats because of the extremely low solubility of DTS (0.000908 mg/mL). In addition, as reported in several studies [16,53,54], the agglomeration of drug particles and the shape of closely packed clusters in the tissue at the injection site may drastically decrease the surface area, thus hindering drug dissolution in the gastrocnemius muscle tissue. The increase in surface free energy due to the rapid absorption of the aqueous suspension vehicle has been reported to cause drug aggregation at the injection site. After reaching C_max_, the plasma concentration of DTS gradually decreased, maintaining over 0.4 ng/mL in the plasma even after 42 days, and fell below the LOQ after 8 weeks. The half-lives (T_1/2α_ and T_1/2β_) for DTS-NS were 5.12 and 9.94 days, respectively, indicating a prolonged duration of drug action. IM-administered NS systems are aggregated in the administered tissue and slowly dissolved, and then steadily absorbed into the blood stream or lymphatic circulation. According to the literature, some fraction of NSs could be uptaken by immune cells, including macrophages at injection sites, and then enter the lymphatic capillaries [10,55]. Drug molecules, having entered systemic circulation, might undergo corresponding metabolism and elimination processes, as DTS absorbed in the gastrointestinal tract. DTS has been reported to be extensively metabolized by the CYP3A4 and CYP3A5 isoenzymes, producing 4′-hydroxydutasteride, 6-hydroxydutasteride, and 6,4′-dihydroxydutasteride. Then, the drug itself and its metabolites (>90%) are excreted mainly in feces [6,56]. In contrast, following the IV bolus administration of the DTS solution, the drug was rapidly distributed and cleared from the bloodstream, with the elimination T_1/2α_ and T_1/2β_ at 0.04 and 0.39 days, respectively. The bioavailability of DTS following the IM injection of NS was determined to be approximately 67%. The remaining (approximately 34%) drug is thought to be still located at the injection site until day 56 or was removed through metabolism or degradation at the injection site. This prolonged delivery of DTS using the NS system, which is comparable to that of the previously reported PLGA microsphere system [8], is advantageous for maintaining therapeutic drug levels over an extended period while minimizing the risk of concentration-related adverse effects. In addition, the novel NS system can be fabricated with a simple and robust fabrication process, providing little to no yield loss, low batch-to-batch variability, and no use of harmful organic solvents. To provide a more continuous and consistent pharmacokinetic profile, further formulation studies including examinations of drug concentration adjustments and DTS particle size control, as well as physicochemical stability tests, are required.

### 3.7. Histopathological Observation of Injection Site Following IM DTS-NS Injection

When water-insoluble drug crystals are administered intramuscularly (IM) or subcutaneously (SC), drug aggregates/depots typically form at the injection site, leading to a localized inflammatory response [16,20,53,54,57]. Upon the introduction of a foreign substance into the body, acute inflammatory reactions occur. This reaction involves the recruitment of inflammatory mediators such as cytokines (TNF-α, IL-1β, and IL-6) and chemokines (MCP-1 and IL-8), as well as inflammatory cells such as neutrophils and macrophages to the inflammation site. Macrophages engage in phagocytosis to remove foreign substances. Previous studies have shown that particles ranging from 0.1 to 10 μm are subject to macrophage phagocytosis [16,58,59]. If inflammatory mediators are not cleared during the inflammatory phase, the foreign material is encapsulated by fibroblasts, platelets, and collagen, resulting in its isolation by a collagen capsule [60]. Persistent inflammation at the injection site can cause pain, redness, swelling, and discomfort, resulting in reduced patient compliance. Thus, the evaluation of local inflammatory responses is essential for the development of LAI systems [61,62].

**Figure 8:**
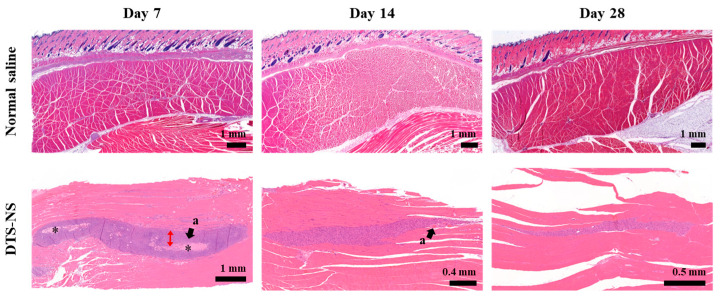
Histological examination of gastrocnemius muscles stained with hematoxylin and eosin (H&E) following intramuscular (IM) injection of negative control (normal saline) or DTS-NS in rats. Note: The depot formed by injection of DTS-NS is indicated by (*). Red double arrows indicate fibroblastic bands, and (a) denotes angiogenesis.

The gastrocnemius muscle tissues injected with normal saline or DTS-DS were further observed histologically using H&E-stained gastrocnemius muscle tissues (Figure 8). The dose of IM DTS-NS was equivalent to that used for the pharmacokinetic evaluation. Injection of normal saline did not cause the infiltration of inflammatory cells or tissue responses in the surrounding tissues during the experimental period. In contrast, noticeable inflammatory lesions were observed in the gastrocnemius muscle tissues injected with DTS-NS. On the seventh day after injection, a drug aggregate-containing depot was observed, along with the infiltration of inflammatory cells, desmoplastic reaction, and fibrosis around the depot. This granulomatous inflammatory reaction against drug particles is commonly observed when drug particles are injected. According to Darville et al., IM-injected paliperidone palmitate crystals (~1000 nm) elicit an injection site reaction consisting of acute inflammation, followed by a chronic inflammatory response [21,53]. A subsequent chronic inflammatory reaction was characterized by the infiltration of inflammatory cells derived from the blood, the phagocytosis of drug crystals by macrophages, granulation, and a dense inflammatory envelope. On the other hand, on the 14th day of observation, the inflammatory response was dramatically reduced, and only partial angiogenesis was observed, with no visible drug depot. Additionally, there was a marked reduction in the area of inflammatory cell distribution compared with that on day 7 (Figure 8). These histopathological trends correlated with drug absorption and pharmacokinetic profiles (Figure 8). On day 7, the presence of the drug depot and the numerous surrounding inflammatory cells indicated that drug release from the depot was accelerated by macrophage phagocytosis, given that the nanoscale size of the DTS-NS made them susceptible to phagocytosis [63,64]. The T_max_ was determined to be 8.40 days. On day 14, the drug depot was no longer visible, suggesting that a substantial amount of the injected DTS had been released and the plasma concentration of DTS had decreased. By day 28, the inflammatory response had largely resolved with only minimal inflammatory cells remaining. These results indicate that the reaction associated with DTS-NS does not progress to a chronic stage but rather that the inflammatory lesions caused by the reaction are undergoing recovery.

## 4. Conclusions

A novel DTS-loaded NS system was successfully prepared using a dual-centrifugation-based wet bead-milling technique with Tween 80 as the suspending agent. DTS-NS exhibited a rod/rectangular shape and nanoscale particle size (324 nm) with weakened drug crystallinity compared with that of the drug powder. In a pharmacokinetic study in rats, the IM injection of DTS-NS provided an extended drug concentration–time profile of over 56 days, with an elimination half-life of 9.94 days. Moreover, local foreign body reactions, including granulomatous inflammatory reactions caused by the IM injection of DTS-NS, were markedly alleviated after 14 days and mostly recovered by day 28 post-dosing. We expected that the NS system would be a favorable long-acting delivery system for DTS with a protracted pharmacokinetic profile and tolerable local inflammation.

## Figures and Tables

**Table 1:** Pharmacokinetic parameters of DTS in plasma following IM administration (5 mg/kg as DTS) of DTS-NS and IV administration (0.2 mg/kg as DTS) of DTS solution in rats. Data are presented as means ± SD (n = 5).

Parameters	DTS-NS	IV Bolus
Dose (mg/kg)	5	0.2
AUC_0–last_ (ng·day/mL)	110.99 ± 30.26	6.63 ± 1.32
AUC_0–inf_ (ng·day/mL)	112.97 ± 33.50	6.84 ± 1.41
C_max_ (ng/mL)	8.06 ± 0.45	17.02 ± 3.24
T_max_ (day)	8.40 ± 3.13	
T_1/2α_^a^ (day)	5.13 ± 2.48	0.04 ± 0.00
T_1/2β_^b^ (day)	9.94 ± 4.06	0.39 ± 0.07
AUC_0–last_/Dose (ng·day/mL/mg)	55.50 ± 15.13	82.92 ± 14.75
Bioavailability (%) ^c^	66.96 ± 18.26 ^c^	

^a^ Time required for plasma concentration to decline by 50% during the distribution phase. The distribution rate constant (α) was calculated using data within the time range from T_max_ to 21 days post-administration. The distribution half-life (T_1_/_2α_) was calculated as T_1_/_2α_ = 0.693/α. ^b^ Time required for plasma concentration to decline by 50% during the elimination phase. The elimination rate constant (β) was calculated using data within the time range from 21 days post-administration to the end of the observation period. The elimination half-life (T_1_/_2β_) was calculated as T_1_/_2β_ = 0.693/β. ^c^ Absolute bioavailability was calculated by multiplying the area under the concentration–time curve (AUC) for the intramuscular (IM) route by the intravenous (IV) dose, dividing by the AUC for the IV route multiplied by the IM dose, and multiplying the result by 100%.

## Data Availability

No new data were created or analyzed in this study. Data sharing is not applicable to this article.

## Supplementary Materials

The following supporting information can be downloaded at: https://www.mdpi.com/article/10.3390/nano14221781/s1, Table S1: Physicochemical stability of DTS-NS at 25 °C. Data are presented as means ± SD (n = 3).
