# Supplementary material for: Design of Nanocrystalline Suspension of Dutasteride for Intramuscular Prolonged Delivery
Source: Nanomaterials (Basel). 2024 Nov 5;14(22):1781. doi: 10.3390/nano14221781 (PMC11597831; doi:10.3390/nano14221781)
Supplement: Supplementary file 1 [file nanomaterials-14-01781-s001.zip › nanomaterials-3251730-supplementary.pdf]

nanomaterials

Supplementary Materials

Design of Nanocrystalline Suspension of Dutasteride for Intramuscular Prolonged Delivery

Min Young Jeong †, Doe Myung Shin †, Min Kyeong Kwon, Ye Bin Shin, Jun Soo Park, In Gyu Yang, Jin Hyuk Myung, Dong Geon Lee, Gi Yeong Lee, Chae Won Park, Ji Won Yeo, Myoung Jin Ho, Yong Seok Choi * and Myung Joo Kang *

College of Pharmacy, Dankook University, 119 Dandae-ro, Dongnam-gu, Cheonan 31116, Chungnam, Republic of Korea; jmy951207@dankook.ac.kr (M.Y.J.); sja9370@dankook.ac.kr (D.M.S.); 72220309@dankook.ac.kr (M.K.K.); shinyb0921@dankook.ac.kr (Y.B.S.); dkjsp0434@dankook.ac.kr (J.S.P.); inq72220320@dankook.ac.kr (I.G.Y.); blueboy0216@dankook.ac.kr (J.H.M.); ldg4337@dankook.ac.kr (D.G.L.); eden1116@dankook.ac.kr (G.Y.L.); 1506chaewon@dankook.ac.kr (C.W.P.); yeo.jiwon13@dankook.ac.kr (J.W.Y.); butable@dankook.ac.kr (M.J.H.)

* Correspondence: analysc@dankook.ac.kr (Y.S.C.); kangmj@dankook.ac.kr (M.J.K.)

† These authors contributed equally to this work.

Table S1. Physicochemical stability of DTS-NS at 25 °C. Data are presented as means ± SD (n = 3).

Duration of storage	Content (%)	Particle size (nm)	Polydispersity Index (PDI)	Zeta potential (mV)
Initial	99.0 ± 0.6	376.4 ± 4.9	0.20 ± 0.01	−11.1 ± 0.5
4 weeks	97.8 ± 0.5	403.6 ± 3.2	0.19 ± 0.04	−14.1 ± 1.2

Citation: Jeong, M.Y.; Shin, D.M.; Kwon, M.K.; Shin, Y.B.; Park, J.S.; Yang, I.G.; Myung, J.H.; Lee, D.G.; Lee, G.Y.; Park, C.W.; et al. Design of Nanocrystalline Suspension of Dutasteride for Intramuscular Prolonged Delivery. *Nanomaterials* **2024**, *14*, x. https://doi.org/10.3390/xxxxx

Academic Editor(s): Name

Received: 25 September 2024
Revised: 30 October 2024
Accepted: 2 November 2024
Published: date

Copyright: © 2024 by the authors. Licensee MDPI, Basel, Switzerland. This article is an open access article distributed under the terms and conditions of the Creative Commons Attribution (CC BY) license (https://creativecommons.org/licenses/by/4.0/).
